# The National Institute on Drug Abuse Summer Research Internship Program: Building a Diverse National Scientific Workforce

**DOI:** 10.1038/s41386-022-01377-3

**Published:** 2022-07-28

**Authors:** Albert H. Avila, Jason H. Weixelbaum, Wilson M. Compton

**Affiliations:** 1 National Institute on Drug Abuse, Bethesda, MD USA; 2 Rose Li & Associates, Chevy Chase, MD USA

**Keywords:** Preclinical research, Outcomes research

## Introduction

Each year, undergraduates in the National Institute on Drug Abuse (NIDA) Summer Research Internship Program (SRIP) arrive in substance use disorder (SUD) research labs across the United States to foster their careers and learn about scientific research. They may not realize it, but these interns are helping to build our future national scientific workforce and contribute to diversity in science, technology, engineering, and mathematics (STEM). The U.S. National Academies of Sciences reported that ~3.4 million technical jobs would be open in 2022, and the national workforce needs to be prepared to fill these essential positions. The current population of scholars entering the workforce needs scientific training to address critical questions impacting health. At the intersection of student, institutional, and national needs, NIDA’s SRIP plays a critical role. The goal of this program is to promote diversity, equity, inclusion, and accessibility (DEIA) in biomedical science careers as part of a broader approach to support STEM education. As a model program, we find that it shows great promise in developing and retaining talented students.

Founded in 1997, NIDA SRIP has funded hundreds of undergraduates to work with investigators across United States. In 2021, NIDA received over 500 applications and matched 83 interns to research sites, compared to an average of 60 per year in 2013–2020. The program’s growth testifies, not only to serving the needs of students, institutions, and society, but also to the success of the model on which the program is based [1, 2]. Growth is also consistent with a 2021, Presidential Executive Order, which called for increased paid internships along with a whole-of-government priority on DEIA in these internships.

Substantial research demonstrates that summer research internships increase retention of talented students in STEM. One study showed undergraduate engagement in research programs *doubled* student interest in pursuing graduate degrees and further research endeavors [3]. Other scholars found that summer research programs contribute to student motivation to pursue science careers [4]. Students themselves score summer research internships highly in their decisions to pursuing a career in science, particularly in biomedical sciences [5, 6].

Research also signifies the importance of summer research internships in promoting DEIA goals. Deployment of institutional resources for recruitment and retention of diverse scholars have been widely identified as best practices [7]. By deliberately promoting diversity, STEM programs can create more inclusive career environments [8]. Mentoring experiences, typical in summer internships, present key opportunities for retention of diverse students [9, 10] and engagement in research initiatives can specifically reduce disparities in STEM [11].

SRIP serves both pedagogical and social goals. As for pedagogical goals, the student receives an opportunity to conduct SUD research to gain real-world biomedical, behavioral, translational, clinical, or epidemiological experience. An internship may include laboratory experiments, data collection and analysis, experimental design, coursework, patient interviews, and manuscript preparation, among other opportunities. Interns typically deliver presentations on their research at the conclusion of the program and may attend weekly seminars on research and career development. SRIP fulfills social goals with multi-directional impact: build and increase diversity in the STEM workforce, expose under-resourced students to research, and serve as a bridge to other programs and graduate school. Since other research programs are available to undergraduates, SRIP focuses on providing internships to students with limited exposure to previous research experiences. Meeting the goals of this program comes with several challenges; the COVID-19 pandemic forced SRIP to skip 2020 and to migrate to a virtual format in 2021, which led to the program offering in-person and virtual experiences going forward. There are also occasional individual challenges, such as a family or personal crisis, handled on an individual basis.

**Fig. 1:**
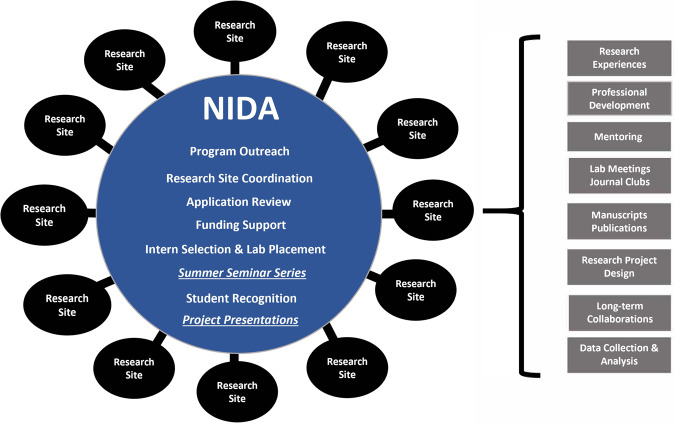
Hub (NIDA) and spokes (research sites) model of the NIDA Summer Research Internship Program.

The NIDA SRIP uses a hub and spoke model where NIDA “hub” and research sites “spokes” work together (Fig. 1). NIDA advertises SRIP to prospective interns through social media platforms, newsletters, and email distribution lists, including minority-serving institution (MSI) outreach. Intern selections are based on career goals, interest in SUD research, site preference justification, and program priorities. It is possible that applicants with high GPAs or several previous research experiences are not selected, as the program prioritizes students with limited research experience. Once confirmed, interns are matched with principal investigators (PIs) at various research sites across the country. NIDA contacts all NIDA-funded grantees with an opportunity to register their research site and mentor interns. All sites are included in the program guide, which interns use for site choices. Interns are matched based on their site justifications. All internships are paid, and 2021 interns received $15 an hour. Cost per intern ranges from $10 to 11,000 which NIDA funds (2021 funding support per intern breakdown: Summer salary support: $4800; Housing subsidy (if applicable): $2500; Travel subsidy (if applicable): $500; Director Supervisor Honorarium: $1000; Institutional Indirect Costs $3000–$4500. There is no standard regional housing set aside; however, on rare exceptions, an alteration in the housing subsidy can be provided for high-cost locations. Interns are paid at federal minimum wage levels across all research sites).

The NIDA hub offers interns seminar courses to expose them to various research areas and resources. These include seminars on basic, clinical, and epidemiological SUD research, the NIH RePORT database, an overview of graduate school, and other career development information. There is also an opportunity for interns to virtually present their research in a SRIP showcase, where interns interact with their peers and share work on their respective projects.

The institutional spokes provide interns research opportunities, training in data collection, reporting, analysis, experimental design, and professional development opportunities (Fig. 1). By working on projects and collaborating with established professionals who mentor students and help them build networks and research expertise, SRIP helps students acculturate to the scientific profession. Nicholas Corriea, former NIDA intern, explained: “I was exposed to many aspects of research…from engaging in dialog with patients in follow-up interviews to assessing counselor sessions for fidelity and providing ongoing feedback…[which] will prove to be valuable in my professional career and work to enhance my application to MD programs in the future.”

## Benefits to students

Students are a major beneficiary of summer internship programs which serve as an important part of STEM career paths. The concept of the “STEM Pipeline” has both its proponents and critics [12]; regardless, each spoke provides transformative opportunities for students, which they may not otherwise experience. These opportunities for interns not only include research exposure, but also building resumes, establishing relationships with mentors, graduate enrollment at universities, and employment at labs where they served. Celia Grace Allred said her SRIP mentor “got me in touch with other faculty for the sole purpose of discussing my graduate school plans and is continuing to help me with my applications.”

DEIA efforts are more than just adding to professional experience. Students also have valuable opportunities to build networks and confidence which can create multiplier effects for diversifying STEM-related fields. NIDA stresses that the SRIP is open to all students and acts as a bridge to other training programs. Lester Rodriguez Santos, another former intern, explained: “While [in SRIP] at Penn, my mentor helped me set up a visit to the NIDA IRP [Intramural Research Program] so that I could inquire about continuing work in the lab. It is because of this meeting I was able to attain a postbaccalaureate fellowship.” Santos says this experience helped him pursue a PhD in neuroscience.

**Fig. 2:**
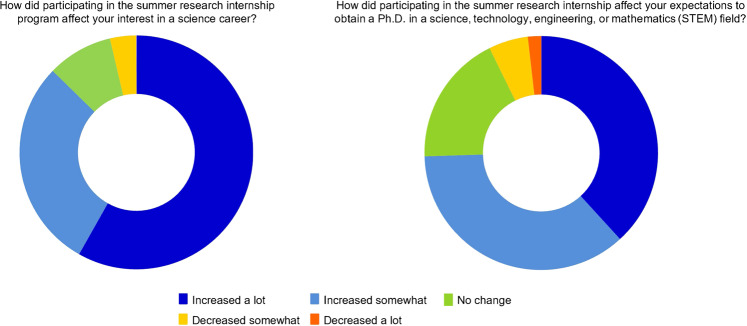
Evaluation responses from NIDA Summer Research Internship Program interns in 2021.

Interns are asked to provide feedback to assess the effectiveness of SRIP for encouraging students to pursue career opportunities in STEM. NIDA typically makes four attempts to contact interns to provide feedback about their SRIP experience. Various data has been collected since 2013 when NIDA SRIP transitioned to an electronic records system. Feedback from 2021 interns (*n* = 55, 66% response rate) demonstrate a broad majority of students increased both their interest in advancing in the profession and in obtaining higher degrees in the field. Over 90% of the responding students report an increased interest in STEM as a result of this research experience, and over 70% of interns report an increased expectation in obtaining a PhD in a STEM field (Fig. 2). These data suggest the program’s positive impact and the potential reach these early research experiences may have on URM students and students in general.

## Benefits to institutions

A clear benefit to universities, laboratories, and other research sites are intern research contributions. Each spoke is driven by the need for a steady stream of low-cost, high performing interns that can quickly learn specific tasks, collect and/or report as much data as possible in a short period, and sustain various research endeavors for extended periods of time [13, 14]. Trained interns serve as a resource for data collection and contributions to publications.

However, the economics of a steady stream of interns are not the only benefit to collaborating institutions. Harder to measure is the beneficial impact of interns on the larger learning community and culture. Interns interacting with researchers and graduate students on sustained, intensive, and novel research projects have the potential to add value to institutions more broadly and contribute to the scientific diversity of the environment. One mentor commented: “Summer interns in our lab increased the visibility of our NIDA-funded substance use disorders research at our university.”

Dr. Linda Cottler, Professor of Epidemiology and Associate Dean of Research at University of Florida (UF) has mentored 27 NIDA SRIP scholars since 2003. She noted that a former intern applied to the UF Epidemiology PhD program and now works with her; others have applied to other UF graduate programs. “It is a wonderful program that PIs should take advantage of…these students are enthusiastic and have a lot to offer.”

Participating institutions also benefit from NIDA’s goal of increasing DEIA. Diversifying learning communities do not just benefit underrepresented groups, but also overall institutional culture [15]. Students from different backgrounds add new perspectives to research sites, and studies show this can increase innovation and build broader connections to local communities [16]. Undergraduates also bring a sense of excitement and are eager to contribute to the research program.

**Fig. 3:**
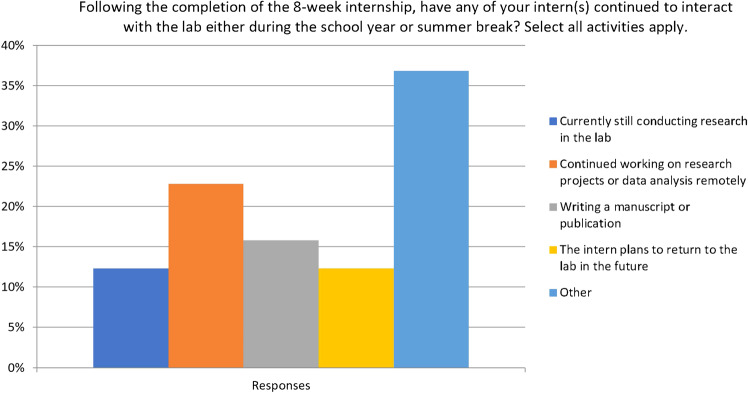
Feedback responses from NIDA Summer Research Internship Program mentors from 2018 to 2021.

According to feedback from past mentors of the program from 2018 to 2021 (*n* = 72, 39 percent response rate), more than 30 percent report that their summer intern continued to conduct research with them after the formal 8-week program (Fig. 3) Since this question only allowed investigators to select one option, comments that included a combination of answers were listed as “other.” These combinations included continued intern research in the lab, working remotely on a different project, correspondences to discuss co-authorship of manuscripts, future career interests, request for letters of recommendation, assistance with graduate school applications, and discussion of future research positions. More than 15 percent of mentors report working on a publication related to their research with the intern. Another 15 percent of mentors report an intention to hire their intern to work in their lab. Other data shows more than 43 percent of students matriculate to graduate school or medical school at the university where they completed their internship or at another university. There is no expectation that interns stay in touch with the lab after the program ends; however, anecdotal evidence shows many interns continue work with mentors on publications, research, and in graduate school.

## Benefits to society

NIDA’s SRIP model is meant not just to facilitate research, but to improve and diversify the nation’s STEM workforce in anticipation of future needs. NIDA is heavily invested in diversifying the biomedical research workforce and seeks to continue to partner with research institutions and other stakeholders to expand the available opportunities for diverse populations. In addition to currently unfilled technical positions, U.S. STEM jobs are expected to grow by 853,600 in the next decade [17]. Workforce needs are changing, and preparations should be made to address potential challenges and shortages. Research shows that sustained focus on DEIA programs can have a demonstrable beneficial effect on increasing equity [18].

Graduates of SRIP are a testament to its success. Dr. Dionna Williams, Associate Director of Clinical Research and Director of the Clinical and Translational Research Core at Harvard University’s Wyss Institute, credits the program for her path: “The NIDA Summer Program changed my entire career trajectory by exposing me to the world of public health through clinical research.”

## Reproducing the model

The Hub and Spoke model espoused by NIDA’s SRIP can be reproduced, with the goal of multiplying beneficial impacts on stakeholder groups. The model helps expand the number of research opportunities available, share administrative burdens, expose students to the breadth of research areas and opportunities available across the U.S., and, most importantly, maximize resources and opportunities. NIDA’s experience demonstrates that with devoted resources and leaders, it can build long-term capacity to expand its reach.

## Conclusions

SRIP is entering its 25th year, which is evidence of its durability and success. Student interest and applications have increased over time and broader institutional cues from the White House and elsewhere demonstrate a demand for work that NIDA supports. However, challenges remain. Growth in STEM participation by underrepresented communities continues to be uncertain and uneven [19]. More effort is needed to address stubbornly entrenched education disparities facing students from diverse backgrounds pursuing a career in STEM. These internships provide students with real life work experience and drive opportunities for both universities and the Nation, leading to a win-win-win.

Research shows that programs like NIDA’s help with student recruitment and retention in STEM fields [20]. Much is written of STEM pipeline “leakage” and how to address it [21]. Attrition rates of low-income and underrepresented groups in STEM remain problematic [22–24]. While more data is needed on the long-term effects of NIDA’s program, this model has benefits beyond a single summer. If every undergraduate student were provided research opportunities, application of this model could not only reduce student attrition rates, but also prepare the biomedical research workforce to address future challenges [25]. To fully benefit the U.S., future innovators should be engaged, have a sense of belonging, and be trained to tackle tomorrow’s problems.

This study comes with an important caveat. We are not comparing results of NIDA’s SRIP to other programs. We recognize that there is more data to collect; however, there is research to suggest that NIDA’s model has a positive impact and could be reproduced to multiply such impacts for students, institutions, and the national workforce by other agencies or institutions coordinating off-site internship programs.

No matter how NIDA or other similar programs choose to continue, the need to grow, improve, and diversify our research workforce will continue to be important. The NIDA Hub and Spoke model can serve as a platform for agencies that might not have all the components in house, but are willing to partner with universities and research institutes to maximize resources. What is clear is the need for a versatile, collaborative, diverse, and more experienced workforce to take on 21st century challenges. From the complexities of SUDs and the COVID-19 pandemic, to the existential threat of climate change, summer internship programs have an outsized opportunity to make a long-lasting positive impact and leave a stronger, inclusive, and more diverse research community for the next generation.

## Disclaimer

The views expressed in this paper are those of the authors and do not necessarily represent the views of the National Institute on Drug Abuse, the National Institutes of Health, or the U.S. Department of Health and Human Services.

## References

[1] National Science Board. The Skilled Technical Workforce: Crafting America’s Science & Engineering Enterprise. 2019. https://www.nsf.gov/nsb/publications/2019/nsb201923.pdf.

[2] National Academies of Sciences, Engineering, and Medicine. Building America’s Skilled Technical Workforce. Washington, DC: The National Academies Press; 2017. 10.17226/23472.

[3] Hathaway R, Nagda B, Gregerman S. The Relationship of Undergraduate Research Participation to Graduate and Professional Education Pursuit: An Empirical Study. J Coll Stud Dev. 2002;43. https://www.researchgate.net/publication/234625388_The_Relationship_of_Undergraduate_Research_Participation_to_Graduate_and_Professional_Education_Pursuit_An_Empirical_Study.

[4] Ghee M, Keels M, Collins D, Neal-Spence C, Baker E (2016). Fine-tuning summer research programs to promote underrepresented students’ persistence in the STEM pathway. CBE Life Sci Educ.

[5] Howell LP, Wahl S, Ryan J, Gandour-Edwards R, Green R (2019). Educational and career development outcomes among undergraduate summer research interns: a pipeline for pathology, laboratory medicine, and biomedical science. Acad Pathol.

[6] Tenenbaum LS, Anderson MK, Ramadorai SB, Yourick DL. High school students’ experience with near-peer mentorship and laboratory-based learning: in their own words. J STEM Educ Innov Res. 2017;18:5–12. https://www.jstem.org/jstem/index.php/JSTEM/article/view/2185.

[7] Peek ME, Kim KE, Johnson JK, Vela MB (2013). URM candidates are encouraged to apply: a national study to identify effective strategies to enhance racial and ethnic faculty diversity in academic departments of medicine. Acad Med.

[8] Ahmad AS, Sabat I, Trump-Steele R, King E. Evidence-based strategies for improving diversity and inclusion in undergraduate research labs. Front Psychol. 2019;10:1305. https://www.frontiersin.org/articles/10.3389/fpsyg.2019.01305/full. 10.3389/fpsyg.2019.01305 PMC6611382 31316412

[9] Freeman BK, Landry A, Trevino R, Grande D, Shea JA (2016). Understanding the leaky pipeline: Perceived barriers to pursuing a career in medicine or dentistry among underrepresented-in-medicine undergraduate students. Acad Med.

[10] Linn M, Palmer E, Baranger A, Gerard E, Stone E (2015). Undergraduate research experiences: Impacts and opportunities. Science.

[11] Schultz PW, Hernandez PR, Woodcock A, Estrada M, Chance RC, Aguilar M, et al. Patching the pipeline: Reducing educational disparities in the sciences through minority training programs. Educ Eval Policy Anal. 2011;33. 10.3102/0162373710392371. 10.3102/0162373710392371 PMC3839574 24285910

[12] Cannady MA, Greenwald E, Harris KN. Problematizing the STEM Pipeline Metaphor: Is the STEM Pipeline Metaphor Serving Our Students and the STEM Workforce?. Sci Ed. 2014;98:443–60. https://onlinelibrary.wiley.com/action/showCitFormats?doi=10.1002%2Fsce.21108.

[13] Maertz, C Stoeberl P, Marks J. Building successful internships: Lessons from the research for interns, schools, and employers. Career Dev Int. 2014;19. 10.1108/CDI-03-2013-0025.

[14] Crowell TL (2018). Academic internships: to take or not to take? Students’ assessments of public health fieldwork. Pedagog Health Promotion.

[15] Martinez-Acosta VG, Favero CB. A discussion of diversity and inclusivity at the institutional level: the need for a strategic plan. J Undergrad Neurosci Educ. 2018;16:A252–60. https://www.ncbi.nlm.nih.gov/pmc/articles/PMC6153014/. PMC6153014 30254540

[16] Salto LM, Riggs ML, Delgado De Leon D, Casiano CA, De Leon M. Underrepresented minority high school and college students report STEM-pipeline sustaining gains after participating in the Loma Linda University summer health disparities research program. PLOS ONE. 2014;9:e108497. https://journals.plos.org/plosone/article?id=10.1371/journal.pone.0108497. 10.1371/journal.pone.0108497 PMC4177228 25250695

[17] Sargent JF Jr. The U.S. science and engineering workforce: Recent, current, and projected employment, wages, and unemployment (CRS Report R43061). Washington, DC: Congressional Research Service; 2017. https://fas.org/sgp/crs/misc/R43061.pdf.

[18] Estrada M, Burnett M, Campbell A, Campbell PB, Denetclaw WF, Gutiérrez CG, et al. Improving underrepresented minority student persistence in STEM. CBE Life Sci Educ 2016;15. 10.1187/cbe.16-01-0038. 10.1187/cbe.16-01-0038 PMC5008901 27543633

[19] Pew Research Center. STEM Jobs See Uneven Progress in Increasing Gender, Racial and Ethnic Diversity. 2021. https://www.pewresearch.org/science/2021/04/01/stem-jobs-see-uneven-progress-in-increasing-gender-racial-and-ethnic-diversity/.

[20] Allen-Ramdial SA, Campbell AG. Reimagining the pipeline: advancing STEM diversity, persistence, and success. BioScience. 2014;64:612–8. https://academic.oup.com/bioscience/article/64/7/612/2754151. 10.1093/biosci/biu076 PMC4282132 25561747

[21] Van den Hurk A, Meelissen M, van Langen A (2019). Interventions in education to prevent STEM pipeline leakage. Int J Sci Educ.

[22] National Academies of Sciences, Engineering, and Medicine. Minority Serving Institutions: America’s Underutilized Resource for Strengthening the STEM Workforce. Washington, DC: The National Academies Press; 2019. 10.17226/25257.

[23] Chen X. STEM Attrition: College Students’ Paths into and out of STEM Fields. Statistical Analysis Report. NCES 2014-001. Washington, DC: National Center for Education Statistics; 2013.

[24] Hudson JK, Hudson BG (2019). Aspirnaut: a rural high school pipeline to increase diversity in STEM. Nat Rev Nephrol.

[25] Business-Higher Education Forum & National Science Foundation. Reskilling America’s Workforce: Exploring the Nation’s Future STEM Workforce Needs. 2018. https://www.bhef.com/publications/reskilling-america%E2%80%99s-workforce-exploring-nations-future-stem-workforce-needs.

